# Signalling Molecules in the Urothelium

**DOI:** 10.1155/2014/297295

**Published:** 2014-08-10

**Authors:** Michael Winder, Gunnar Tobin, Daša Zupančič, Rok Romih

**Affiliations:** ^1^Department of Pharmacology, Sahlgrenska Academy, University of Gothenburg, Medicinaregatan 13, 405 30 Gothenburg, Sweden; ^2^Institute of Cell Biology, Faculty of Medicine, University of Ljubljana, Vrazov trg 2, 1000 Ljubljana, Slovenia

## Abstract

The urothelium was long considered to be a silent barrier protecting the body from the toxic effects of urine. However, today a number of dynamic abilities of the urothelium are well recognized, including its ability to act as a sensor of the intravesical environment. During recent years several pathways of these urothelial abilities have been proposed and a major part of these pathways includes release of signalling molecules. It is now evident that the urothelium represents only one part of the sensory web. Urinary bladder signalling is finely tuned machinery of signalling molecules, acting in autocrine and paracrine manner, and their receptors are specifically distributed among different types of cells in the urinary bladder. In the present review the current knowledge of the formation, release, and signalling effects of urothelial acetylcholine, ATP, adenosine, and nitric oxide in health and disease is discussed.

## 1. Introduction

**Figure 1:**
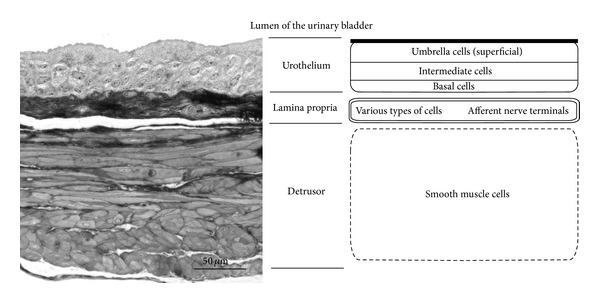
Urinary bladder wall of the mouse. An image of a semithin epon section and a schematic representation of the urinary bladder wall. The urothelium is composed of superficial umbrella cells, intermediate cells, and basal cells. Beneath the urothelium there is suburothelial connective tissue termed lamina propria, which contains various types of cells (e.g., fibroblasts, interstitial cells, and myofibroblasts) and afferent nerve terminals. The outermost layer is the detrusor smooth muscle cell layer.

The urothelium is a stratified epithelium that covers the inner parts of the renal pelvis, ureters, and urinary bladder and parts of the urethra. The outermost layer of the urothelium consists of large, flattened so called umbrella cells that are interlocked by tight junctions. The innermost layer of the urothelium consists of smaller basal cells that are separated from the suburothelial lamina propria by a basal lamina (Figure 1). In between the umbrella and basal cells there are intermediate cells. In the urinary bladder of rodents there is 1 intermediate cell layer (Figure 1), whereas in humans there are up to 5 intermediate layers present [1, 2]. The function of the urothelium was long considered only to be a barrier against the bladder urine content. However, today a number of dynamic qualities of the urothelium are well recognised [3, 4]. Several urothelial signalling molecules acting on different receptor subtypes may interact within the urothelium and also modulate afferent neuronal activity and detrusor smooth muscle function.

One significant function of the urothelium is to act as a mechanosensory conductor [3, 5, 6]. Distension of the bladder wall stretches the urothelium [7], which induces the release of adenosine-5′-triphosphate (ATP), as well as a number of other substances such as acetylcholine (ACh) [8] and nitric oxide (NO) [9]. ATP has been mainly associated with the activation of afferent signalling, whereas the significance of acetylcholine and nitric oxide is not fully revealed. However, the interaction of urothelial-derived signalling molecules and sensory fibres is complex and may involve suburothelial interstitial cells [10]. The stimulation of purinergic receptors on afferent nerve terminals is a well-established mechanosensory mechanism [3, 11]. The urothelium can also interact with underlying tissues by release of additional signalling molecules upon activation of urothelial cell surface receptors (Table 1).

Another important implication of the urothelial function is its role in lower urinary tract disorders. For instance, in cyclophosphamide-induced cystitis and in interstitial cystitis/painful bladder syndrome (IC/PBS), the release of urothelial ATP is enhanced and the sensitivity of the afferent nerve terminals is altered [12, 13]. Furthermore, the acetylcholine effect on afferent muscarinic receptors seems to be one important target for drugs used to treat the overactive bladder [14, 15]. These effects, together with the number of signalling molecules produced by the urothelium, in particular ATP, adenosine, and nitric oxide, implicate the involvement of the urothelium in various mechanisms involved in lower urinary tract disorders [16]. Here, the current knowledge of the functional roles of key urothelial signalling molecules, such as acetylcholine, ATP, adenosine, and nitric oxide, is reviewed.

## 2. Acetylcholine

Acetylcholine is a phylogenetically old substance, which more or less all living organisms are able to synthesize [17]. Today two sources of acetylcholine are recognized, neuronal and nonneuronal tissues. While the neuronal transmitter role of acetylcholine was established almost a hundred years ago [18], the role of the nonneuronal system has for long been overlooked. However, lately the importance of the nonneuronal cholinergic signalling in the urinary bladder urothelium has been addressed in research.

### 2.1. Formation of Acetylcholine

According to the classical view, acetylcholine is formed by the enzyme choline acetyltransferase (ChAT) from acetyl coenzyme A and choline, which occurs by the transfer of an acetyl group from acetyl coenzyme A to choline in the nerve terminal [19]. However, in addition to this type of acetylcholine synthesis, the mitochondrial enzyme carnitine acetyltransferase (CarAT) may also contribute to acetylcholine formation [20–22]. In the urothelium, CarAT seems to be the major acetylcholine synthesizing enzyme [23].

Acetylcholine is hydrolysed by either of two structurally similar enzymes in the body, acetylcholinesterase (AChE) and butyrylcholinesterase (BuChE) [24]. In the synaptic cleft, acetylcholine is metabolized by AChE within milliseconds after its release from the nerve, ending into choline and acetic acid. It seems that the urothelium has an afferent innervation and that half of these neurones in the urothelium contain AChE [25]. However, in other types of nonneuronal acetylcholine producing cells, both AChE and BuChE activity have been reported [26]. It is therefore possible that local degradation of urothelial acetylcholine occurs by involving both types of choline esterases.

### 2.2. Release of Acetylcholine

Acetylcholine release may occur either in a vesicular (quantal) or in a nonvesicular (nonquantal) way [27]. The vesicular release is typical for the exocytosis of acetylcholine-containing vesicles in nerve terminals. This exocytosis is induced by the impulse activity, which is dependent on calcium influx via voltage-gated Ca^2+^ channels [28]. The less well-characterized nonvesicular release often occurs independently of impulse activity, either in neurons or in nonneuronal tissues [29]. In the urothelium, where stretch can cause a release of acetylcholine [30], a nonvesicular release of acetylcholine seems to occur, since the vesicular acetylcholine transporter (VAChT) is not present. In the urothelium, the organic cation transporters (OCT1 and OCT3) seem to be of particular importance for the release of acetylcholine [23].

### 2.3. Physiological and Pathophysiological Effects of Acetylcholine

**Figure 2:**
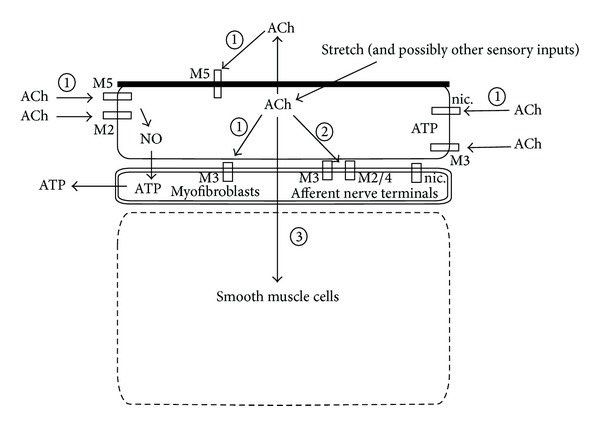
Schematic representation of acetylcholine release and its effects in the urinary bladder wall. Acetylcholine is released from urothelial cells and may target nicotinic (nic.) and muscarinic receptors (M) and thereby modulate ATP and nitric oxide release by the urothelium and possibly also by myofibroblasts (1). Moreover, acetylcholine may also activate nicotinic, M2, M3, and M4 receptors on afferent nerve terminals and therefore modulate sensory signalling (2). Urothelial acetylcholine may also directly affect contraction (3).

Urothelially released acetylcholine can target nicotinic and muscarinic receptors on afferent nerve terminals, myofibroblasts, and detrusor smooth muscle cells as well as cholinergic receptors on urothelial cells (Figure 2) [31]. When instilling oxotremorine methiodide into the bladder of anaesthetized rats, concentration dependant responses occur that are elicited via ATP and nitric oxide [32]. Also, in the pentobarbitone-anaesthetised rat (in which effects via afferent nerves are eliminated), the removal of the urothelium shows that urothelial factors can directly influence the detrusor muscle [33]. In the urothelium, acetylcholine may act on nicotinic as well as on muscarinic receptors (Figure 2) [34–36]. The cholinergic effects may be either excitatory (e.g., via receptors M1, M3, and M5 on nerve terminals or detrusor muscle) or inhibitory (e.g., via receptors M2 and M4 on nerve terminals or detrusor muscle), possibly depending on the intensity of stimulation as indicated by the different effects caused by different concentrations of the muscarinic receptor agonist [37, 38]. The indirect acetylcholine effects via urothelial cells may be induced by the release of a number of substances such as ATP, nitric oxide, and prostanoids [39, 40]. In the rat urothelium, two types of nicotinic receptors are present, *α*7 homomeric and *α*3-containing heteromeric receptors, which inhibit and stimulate bladder reflexes, respectively [37]. Stimulation of the *α*7 receptors and the *α*3-containing receptors with low concentrations of acetylcholine inhibits the release of urothelial ATP [35]. Stimulation with high concentrations of acetylcholine, however, causes the *α*3-containing heteromeric receptors to stimulate the ATP release. Upregulation of urothelial nicotinic receptors has been proposed to contribute to the pathogenesis of detrusor overactivity in bladder outlet obstruction [41].

Urothelial cells express a high density of muscarinic receptors, even higher than the bladder smooth muscle cells, as has been shown in the porcine urinary bladder [42]. Even in the rat and human urothelium, the receptor proteins and mRNAs for all five muscarinic receptor subtypes (M1–M5) occur (Table 1) [34, 43]. In a study on the human urothelium, Bschleipfer et al. [41] reported that the expression pattern of the muscarinic receptor subtypes varies throughout the tissue. While the M1 receptors mainly occur on basal cells, the M2 receptors occur mostly on umbrella cells. Muscarinic M3 and M4 receptors are homogenously distributed while M5 receptors occur with a decreasing gradient from the umbrella to the basal cells. Based on studies of cultured rat urothelial cells, it has been suggested that activation of muscarinic M1, M2, and M3 receptor subtypes may stimulate the release of ATP through the increase of intracellular Ca^2+^ [44, 45]. However, it has also been reported that an effect of urothelial muscarinic receptor stimulation may inhibit the ATP release induced by nicotinic receptor stimulation [22, 35]. Also, muscarinic receptor stimulation may induce the release of nitric oxide [33, 46], which further adds to the complexity of the composite urothelial functions. In addition to the urothelial location, muscarinic receptors are also expressed on afferent nerve terminals (Figure 2) and in the lamina propria on myofibroblast-like cells [47]. The mucosal muscarinic receptors are of the M2 and M3 and possibly also of the M5 subtype [48–50]. These mechanisms may indirectly affect suburothelial nerves [51]. A direct cholinergic effect on the neurons may also occur, since muscarinic M2, M3, and M4 receptors are expressed on murine bladder afferent nerve terminals [52]. While the M2 and M4 subtypes have been suggested to inhibit the signalling, the muscarinic M3 receptors may stimulate the sensory activity (Figure 2).

The release of urothelial acetylcholine has been reported to increase with age in the human bladder [8]. However, the total amount of the muscarinic receptor protein seems to decline [34]. In patients suffering from overactive bladder, the increase in the cholinergic afferent effects has been discussed, namely, that a sensitization of the urothelially acetylcholine-evoked signalling occurs [16]. This is likely to be caused also by upregulation of the cholinergic targets rather than by just increased acetylcholine production. One such target that has been discussed is muscarinic M2 receptors on afferent nerve terminals [53]. Furthermore, in cystitis, the urothelial expression of muscarinic receptors may be changed [48, 54]. In the rat urinary bladder the expression of urothelial muscarinic M1 and M5 receptors is upregulated in the state of inflammation [48]. Activation of the mucosal muscarinic receptors has been reported to be coupled to the release of nitric oxide, and this pathway seems to be altered in the inflamed urinary bladder [33, 46].

Urothelial acetylcholine may thus facilitate and inhibit afferent signalling. Exactly under which circumstances the respective effect that dominates still remains unclear. However, the urothelial cholinergic system may be affected in lower urinary tract disorders. Both the autocrine and the paracrine acetylcholine effects may be part of the chain exerting the changes induced by the pathogenesis.

## 3. ATP

ATP is a multifunctional ubiquitous biological molecule that acts as the primary intracellular energy source for all living cells and also as an extracellular signalling molecule. In the urinary bladder ATP is the main signalling molecule with a pivotal role in bladder fullness sensation and in various bladder disorders [55].

### 3.1. Formation of ATP

ATP is synthesized in an energetically unfavourable phosphorylation reaction in which a phosphate group is added to adenosine diphosphate (ADP). Most of the cell's ATP is produced in the mitochondria via oxidative phosphorylation, while small amounts are also formed in the cytosol via glycolysis. ATP is abundant in the cell cytoplasm (2–5 mM) and higher concentrations of ATP (up to 100 mM) are stored in synaptic vesicles of neurons. Synaptic vesicles also contain other nucleotides such as ADP, adenosine monophosphate (AMP), and guanosine triphosphate (GTP), but at lower concentrations.

Until recently, it was assumed that the only sources of extracellular ATP are cells which are damaged or dying. It is now evident that ATP is released from many cell types including peripheral and central neurons as well as many nonneuronal cell types during mechanical deformation in response to shear stress, stretch, osmotic swelling, hypoxia, and stimulation by various agents [56, 57].

### 3.2. Release of ATP

Precise transport mechanisms involved in ATP release are still under active debate. There is compelling evidence for exocytotic vesicular release of ATP from neurons [58], while several additional mechanisms for ATP release from nonneural cells have been proposed, including transport via connexin or pannexin hemichannels [59–61] and perhaps maxi ion channels, ATP-binding cassette transporters, and P2X_7_ receptor channels [62].

**Figure 3:**
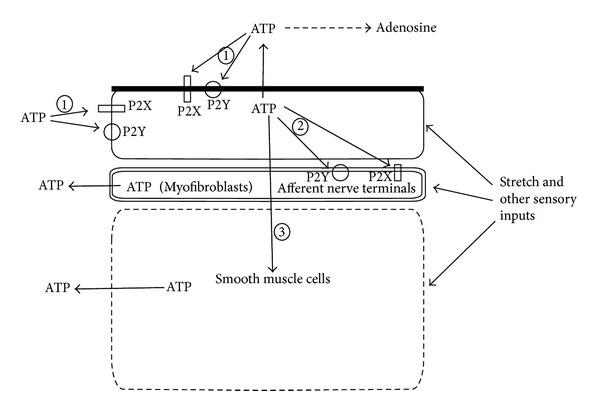
Schematic representation of ATP release and its effects in the urinary bladder wall. Stretch and other sensory inputs (mediators present in the urine and mediators released from nerve processes, urothelial cells, or other compartments of the bladder wall) induce the release of ATP from urothelial cells as well as from myofibroblasts of the lamina propria and from smooth muscle cells of the detrusor. Extracellular ATP can cause increased exocytosis and endocytosis of the umbrella cells (1) via P2X and P2Y receptors; it can signal bladder filling to the CNS (2); and it can lead to detrusor contraction (3). Extracellular ATP can also be degraded into adenosine.

Filling of the urinary bladder stretches the urothelium and thereby activates mechanotransduction pathways, which are likely initiated by increased tension at the apical surface of the umbrella cells. These external mechanical stimuli induce stretch-activated ATP release from urothelial cells [3, 63, 64] (Figure 3). Moreover, ATP release from urothelial cells can also be induced by mediators present in the urine and mediators released from nerve processes, urothelial cells, or other compartments of the bladder wall [45, 63, 65]. The pathways underlying ATP release in the urothelium are mainly attributable to vesicular transport or exocytosis [66] and, to a smaller extend, to pannexin hemichannel conductive efflux [60, 61]. In urothelial cells two additional mechanisms of ATP release were observed: (i) uridine triphosphate- (UTP-) mediated calcium-dependent ATP release and (ii) carbachol-mediated calcium-independent ATP release [45]. Regardless of pathways involved, it is recognised that urothelium is capable of releasing ATP into both mucosal and serosal compartments of urinary bladder wall [3, 65] and thus acts as an important autocrine [66] and paracrine mediator [67], respectively.

### 3.3. Physiological Effects of ATP

The purinergic signalling hypothesis was proposed in 1972 [68], after data had emerged showing that ATP is the transmitter mediating nonadrenergic, noncholinergic neurotransmission in nerves supplying the gut and the urinary bladder [69].

Once it is outside of the cell, ATP in nanomolar concentrations functions as an autocrine/paracrine signal, modulating a broad range of cell and organ functions through activation of purinergic receptors in the cell's plasma membrane. There are two types of purinergic receptors, one selective for adenosine (P1 receptors or AR) and another selective for ATP/ADP (P2 receptors) [62]. Pharmacological and cloning experiments distinguished two families of P2 receptors, that is, P2X and P2Y family. Seven subtypes of P2X receptors (P2X_1_–P2X_7_) [70] and eight subtypes of P2Y receptors (P2Y_1_, P2Y_2_, P2Y_4_, P2Y_6_, P2Y_11_, P2Y_12_, P2Y_13_, and P2Y_14_ isoforms) [71] are currently recognised.

Although some contradictory results are emerging regarding P2 receptor expression in the urinary bladder, it seems that urothelium expresses multiple purinergic receptors, including all 7 P2X receptors as well as P2Y_1_, P2Y_2_, and P2Y_4_ receptors (Table 1) [72, 73]. In the urothelium, ATP is released from both the apical and basolateral urothelial surfaces and can act via P2X_2_ and P2X_3_ receptors present on the urothelial cells to stimulate stretch-induced exocytosis, as well as endocytosis [66] (Figure 3). Exocytosis and endocytosis are regulated in a way that the net effect of stretch is an increase in urothelial surface area [74]. Based on these findings we assume that in urothelial cells exocytosis and endocytosis may regulate the composition of receptors at the plasma membrane of urothelial cells.

It is hypothesized that ATP released from the basolateral surface of the urothelium during bladder filling stimulates P2X_3_ receptors on suburothelial sensory nerve fibres, thus relaying information about the degree of bladder filling to the central nervous system [67] (Figure 3). Consistent with this hypothesis, knockout mice lacking P2X_2_, P2X_3_, or P2X_2_/P2X_3_ receptor subunits can still release ATP from their urothelium, but activation of bladder afferent nerve terminals is significantly decreased and knockout mice showed reduced micturition frequencies and increased bladder capacities [75]. The expression of P2X and P2Y purinergic receptors in nerve fibres and myofibroblasts located near the luminal surface of the bladder [76] and the sensitivity of these cells to ATP suggest that basolateral ATP release from the urothelium may also influence their function. In addition, intercellular communication mediated by gap junctions in myofibroblasts could provide a mechanism for long-distance spread of signals from urothelium to the detrusor muscle cells [77]. Indeed, a recent study demonstrated a paracrine action of ATP released from urothelial cells, which induced detrusor smooth muscle contraction [45] (Figure 3).

The net concentration of ATP in the extracellular space is regulated by ATP release and its breakdown by ectonucleotidases (discussed in Section 4.1). It was proposed that due to ectonucleotidases in the bladder wall half-life of ATP released from basolateral side of urothelium is shortened [65].

### 3.4. ATP Related Urinary Bladder Disorders

Abnormalities in ATP release and in purinergic receptor expression have been noted in numerous studies of human bladder diseases as well as in animal models of bladder pathology [78–84]. These include interstitial cystitis, urinary urgency and incontinence, spinal cord injury-induced bladder dysfunction, detrusor overactivity, and outlet obstruction [78–84]. For example, stretch-activated ATP release from urothelial cells is significantly greater in patients with interstitial cystitis than in healthy individuals [80]. Similar increase in stretch-activated ATP release was observed in the cat model of interstitial cystitis [81] and in cyclophosphamide-induced cystitis in rats and mice [82]. Moreover, upregulation of P2X_2_ and P2X_3_ expression was documented in human interstitial cystitis [78]. Interestingly, reduced extracellular ATP hydrolysis was observed in human detrusor smooth muscle samples from patients with bladder carcinoma, idiopathic bladder instability, and obstructed bladder [83]. It has been demonstrated that extracellular ATP also has tumour-suppressive effects via proinflammatory role and direct cytotoxic function of its receptor P2X_7_ [84–87]. On the other hand, extracellular ATP can promote tumour growth directly by activation of P2 receptors, including P2X_7_, on tumour cells [84, 88–90]. Nevertheless, antineoplastic action of extracellular ATP was documented in urinary bladder cancer cells and P2X_5_ and/or P2Y_11_ receptors may be implicated in this response [79].

## 4. Adenosine

Although more emphasis is placed on the physiology and pathophysiology of ATP, the roles of its breakdown product, adenosine, are also under intense investigation. Adenosine is an endogenous molecule that, besides acting as a component of DNA and RNA, plays a role as a transmitter substance when functioning in the extracellular space [68, 91]. In the urothelium adenosine acts in response to mechanical stimuli, namely, stretches during bladder filling and could modulate sensory afferent function and the contraction of detrusor smooth muscle cells [92].

### 4.1. Sources of Adenosine

The prevailing pathway leading to high extracellular concentrations of adenosine is the extracellular hydrolysis of ATP by ectonucleotidases [93]. Ectonucleoside triphosphate diphosphohydrolases (ENTPDases, CD 39) and ectonucleotide pyrophosphatase/phosphodiesterases (ENPP) hydrolyse ATP and ADP to AMP. 5′-nucleotidases (NT5E, CD73) then further hydrolyse AMP to adenosine [84]. The NT5E is considered as the rate-limiting enzyme in the generation of extracellular adenosine [94]. It should be noted that adenosine is broken down further by adenosine deaminase to inosine and hypoxanthine, which are then removed by the circulation [62].

**Figure 4:**
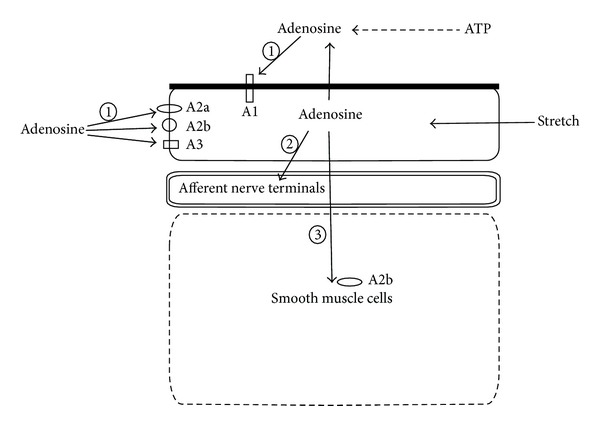
Schematic representation of adenosine release and its effects in the urinary bladder wall. Stretch induces the release of adenosine from luminal and basolateral surfaces of urothelial cells. Extracellular adenosine targets A1 receptors on the apical plasma membrane and A2a, A2b, and A3 receptors on the basolateral plasma membrane of the urothelial cells and causes inhibition of ATP release from urothelial cells (1). Adenosine can also modulate afferent nerve activity (2). Contraction of smooth muscle cell is influenced by adenosine, presumably via A2b receptors (3).

Studies in the mouse urinary bladder have shown a cell-specific expression pattern of ectonucleotidases within the urothelium, lamina propria, blood vessels, and smooth muscle cells and therefore they likely act in a coordinated manner to regulate adenosine availability to purinergic receptors [95]. ENTPDase1, ENTPDase2, ENTPDase3, ENTPDase8, and NT5E achieve stepwise conversion of extracellular ATP to adenosine (Figure 4) in different parts of the urinary bladder wall. To date, ENTPDase3 was shown on membranes of intermediate and basal urothelial cells but not in umbrella cells, and immunofluorescence of ENTPDase8 suggested localization to the urothelium [95]. We believe that the forms and expression patterns of these enzymes in health and disease are likely to affect the response profile of a specific cell.

In addition to adenosine production from ATP and AMP in extracellular space, it was proposed that the urothelium releases adenosine in response to stretch from its luminal and basolateral surfaces (Figure 4). It was shown that adenosine may be produced in significant quantities by the urothelium [96], but the mechanisms of adenosine release have not yet been extensively studied. It is noteworthy to mention that adenosine can also be released from nerves, inflammatory cells, and even blood vessels [97].

### 4.2. Physiological and Pathophysiological Effects of Adenosine

Adenosine exhibits its effects by binding to and activating adenosine receptors (P1 receptors or AR), which are 7 transmembrane plasma membrane receptors, currently divided into four subtypes, namely, the A1, A2a, A2b, and A3 receptors [62, 84, 98]. A1, A2a, and A3 are activated at physiological concentrations of adenosine (30–300 nM), while A2b receptor has relative low affinity for adenosine and therefore requires high levels of adenosine, which may be generated in response to pathological conditions [99, 100].

Because of the various experimental approaches used, it is currently not clear which adenosine receptors are expressed in the urothelium or what is their exact subcellular localization in the urothelial cells [96, 101]. By Western blot analysis, all four adenosine receptors have been confirmed in the urothelium of rabbits, rats, and mice (Table 1) [96]. A1 receptors are prominently localized to the apical plasma membrane of the umbrella cells, whereas A2a, A2b, and A3 receptors are localized intracellularly or in the basolateral plasma membrane of umbrella cells and the plasma membrane of the underlying intermediate and basal urothelial cells [96] (Figure 4). Transcripts for the A1, A2a, and A2b receptors, but not for the A3 receptor, have been detected in human urothelial cells as well as in the human bladder carcinoma T24 cell line [102–104].

In umbrella cells, adenosine has been proposed to modulate exocytosis via the apical plasma membrane [96]. It seems that multiple adenosine receptors may be involved in regulating exocytosis, which was shown by changes in urothelial membrane capacitance [66].

Moreover, by binding to its receptors adenosine can initiate intracellular responses necessary for an appropriate voiding reflex [101]. Adenosine reduces the force of nerve-mediated detrusor contractions by acting predominantly at presynaptic sites of the neuroeffector junctions via the A1 receptor [105]. Following the contractile phase of voiding, ENTPDase1 and NT5E are acting coordinately and rapidly convert ATP to adenosine in order to not only affect cessation of P2X_1_-mediated muscle contraction but also facilitate muscle relaxation through A2b receptors. Relaxation is clearly a prerequisite for accommodating the next filling cycle [106]. Support for this hypothesis comes from studies showing that adenosine receptor A2b is abundantly expressed in detrusor and, further, that adenosine inhibits detrusor contraction [95] (Figure 4). These actions of adenosine are potential modulatory targets for the management of detrusor overactivity [51, 64]. Furthermore, it seems that A1 receptors regulate contractility in healthy and inflamed urinary bladders [107] and adenosine has also been identified as a significant inhibitor of inflammation by acting on A2a receptors [103]. We therefore think that adenosine and its receptors might have a role in inflammation during cystitis and their protective potency should be further studied to improve currently used cystitis treatment. NT5E has been found to be overexpressed in several types of cancer, including bladder cancer. A differential pattern of ectonucleotidases in the more malignant human bladder cancer cells compared with cells derived from an early stage of bladder cancer has been described [108]. Since high levels of NT5E expression were demonstrated in various types of cancer cells, it was proposed that targeting the NT5E may provide an alternative approach to cancer treatment [84].

## 5. Nitric Oxide

Nitric oxide is a small gaseous free radical with a half-life of less than 6 seconds that is generally accepted as one of the nonadrenergic, noncholinergic (NANC) transmitters affecting the bladder [109].

### 5.1. Formation of Nitric Oxide

Nitric oxide is formed in cells when the natural amino acid L-arginine is converted by nitric oxide synthase (NOS). NOS consists of, at least, three distinct isoforms: endothelial nitric oxide synthase (eNOS), neuronal nitric oxide synthase (nNOS), and inducible nitric oxide synthase (iNOS). Two of the isoforms, namely, eNOS and nNOS, are highly dependent on the presence of Ca^2+^ for proper formation of NO, while iNOS is not. Further, eNOS and nNOS are considered to be constitutively expressed while the expression of iNOS occurs upon certain signalling, such as inflammatory signals. This, however, is slightly misguiding as the level of eNOS and nNOS expression also can vary heavily depending on external and internal cell milieu. Expression of NOS has been described in various parts of the bladder tissue. Several reports indicate that formation of nitric oxide can occur in urothelial cells via either iNOS or eNOS, with some reports also indicating the presence of nNOS, mainly in other species than human [110–113]. The expression can be heavily altered by various disorders, especially inflammatory diseases [48, 114, 115], and levels of nitric oxide in urine have been shown to increase in patients with interstitial cystitis [116]. Further, it has been shown that lipopolysaccharide (LPS) treatment can cause upregulation of iNOS in the urothelium [117]. Likewise, it was suggested that urinary bladder lesions can alter the differentiation of superficial urothelial cells, thereby inducing the formation of iNOS in urothelial mitochondria [118]. Many studies point out that iNOS is the important isoform for formation of urothelial nitric oxide in the acute phase of cell damage and early inflammatory response [117, 119]. Nevertheless, Giglio et al. [48] have shown that 60 hours after a single intraperitoneal injection of cyclophosphamide in rats, at a time point when the innate inflammatory response is at its peak, the expression of eNOS seems to be heavily increased in the urothelium and suburothelium. Several other studies have also revealed that the expression of eNOS can vary greatly during certain disease states [113, 120]. Apart from the urothelium, there seems to be several other sources for nitric oxide in the urinary bladder, that is, interstitial cells in the lamina propria [48] and various nerves [121–123]. Even though few studies have shown presence in human urothelium, nNOS and the subsequent formation of nitric oxide in afferent nerve terminals seem to play an important role for normal bladder function as disruption of its gene causes voiding abnormalities in mice [110, 124]. As the levels of eNOS, the levels of nNOS can be altered in the urothelium and suburothelium during certain disease states [125].

### 5.2. Release of Nitric Oxide

Based on its gaseous properties, it has long been assumed that nitric oxide cannot be stored in and therefore cannot be released from vesicles. A recent study in mice has shown that when utilizing a neurotoxin that inhibits vesicular release of neurotransmitters, the release of nitric oxide from the urothelium is not decreased but increased during bladder distension [126]. Likewise, studies performed in spinal cord injured rats show an increased release of urothelial nitric oxide upon botulinum toxin inhibition of vesicular release [82]. Thus, there are several strong indications that neither nitric oxide storage nor release is vesicular.

**Figure 5:**
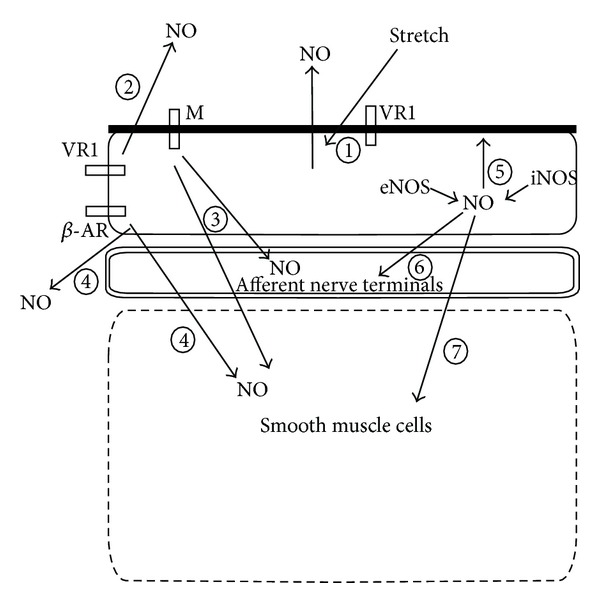
Schematic representation of nitric oxide release and its effects in the urinary bladder wall. Mechanical stretch (1) or activation of vanilloid receptors (2) can cause release of nitric oxide from the urothelium. Mechanically induced release seems to be dependent on the presence of vanilloid receptors (1). Activation of muscarinic receptors (3) or beta-adrenoceptors (4) can cause release of nitric oxide from the urothelium, affecting detrusor contractility directly or indirectly by modulating the activity of afferent nerve terminals located in the lamina propria. While nitric oxide formed from eNOS has been shown to increase urothelial barrier function (5), desensitize afferent nerve terminals (6) and attenuate detrusor contractility (7), nitric oxide formed from iNOS has been shown to cause disruption of barrier function (5), sensitize afferent nerve terminals (6), and facilitate bladder contraction (7).

Various receptors present in the cell surface can play a part in the release of nitric oxide from the urothelium (Table 1). For instance, it has been suggested that nitric oxide can be released from urothelial cells upon mechanical force or stretch (Figure 5), mimicking bladder distension, and that this release is dependent on the presence of vanilloid receptors [127]. In studies utilizing vanilloid agonists, primarily capsaicin and resiniferatoxin, activation of vanilloid receptors present in the urothelium has been shown to cause a Ca^2+^-dependent release of nitric oxide (Figure 5) [128]. Several studies also indicate that nitric oxide can be released from the urothelium upon activation of muscarinic receptors and that this release can directly or indirectly attenuate detrusor contractility (Figure 5) [32, 33]. Characterization of this mechanism has been carried out in bladder strips from healthy and inflamed rat bladders, implicating muscarinic M5 receptors as the subtype primarily involved [46]. Nitric oxide has also been shown to be released from the urothelium in response to noradrenaline [121]. Studies on cultured urothelial cells could show that activation of beta-adrenoceptors can cause a Ca^2+^-dependent release of nitric oxide (Figure 5) [110]. One might therefore assume that treatment of overactive bladder with beta 3-adrenoceptor agonists causes release of nitric oxide that, at least in part, contributes to the relaxatory effect on the detrusor [129]. However, several studies have tried to prove this without finding much evidence for it [130, 131].

### 5.3. Physiological and Pathophysiological Effects of Nitric Oxide

Nitric oxide has been shown to affect afferent nerve signalling, mainly in an inhibitory fashion (Figure 5) [132]. Studies by Pandita et al. [133] using oxyhemoglobin, a nitric oxide scavenger, showed that absence of nitric oxide causes bladder overactivity, possibly via modulation of the threshold for afferent nerve firing. A possible mechanism of action for this is a cGMP signalling pathway modulating high-voltage Ca^2+^ channels, thereby affecting the firing threshold [134]. Cystometrical studies performed on rats have further strengthened the idea of nitric oxide partly exerting its effect on afferent nerve terminals by showing that intravesical administration of a nitric oxide donor increases the time intervals between contractions [135]. In a study performed on anaesthetized rats, intravesical administration of a muscarinic agonist was shown to decrease voiding frequency by affecting the firing of bladder C-fibre afferent nerves [32]. This effect could be abolished by inhibition of NOS, demonstrating a link between activation of urothelial muscarinic receptors and the subsequent release of nitric oxide.

Several studies have been performed in order to outline the effect of nitric oxide on bladder activity during inflammatory bladder disorders. When inducing cystitis by treatment with cyclophosphamide, bladder overactivity is evident. When rats with cyclophosphamide-induced cystitis were treated with an unselective NOS-inhibitor (N^*ω*^-nitro-L-arginine methyl ester; L-NAME), further increase of bladder overactivity could be observed [135, 136]. An important issue to outline is whether the observed effect of nitric oxide during cyclophosphamide-induced cystitis is exerted via modulation of afferent nerve terminals or by acting directly on the detrusor.

Despite the fact that most studies regarding the effects of nitric oxide have looked at its effect on the contractile properties of smooth muscle, studies in various tissues have shown that nitric oxide also can have an important role during the development of inflammation as well as during necrosis/apoptosis and the subsequent loss of barrier function [113, 119, 137, 138]. Common for most of these studies is that is seems to be formation via iNOS that is key for the immunomodulatory effects of nitric oxide, that is, Ca^2+^-independently, that is key for the immunomodulatory effects of nitric oxide [139]. This seems to be true also regarding the urothelium, since it has been shown that prevention of the formation of nitric oxide with a NOS-inhibitor decreases urothelial damage and subsequent inflammation [140]. There are also numerous studies pointing out the involvement of nitric oxide during human and experimental cystitis [114, 115]. Further, regarding bacterial infections causing inflammation, when putting together pieces of information it seems as if nitric oxide plays a key role. In view of the fact that bacterial LPS can cause an increase of iNOS expression in urothelial cells [117], it is possible that the subsequent apoptosis and disrupted barrier function is due to high levels of nitric oxide [119]. Likewise, nitric oxide can play an equally important role regarding barrier function in several noninfectious inflammatory disease states [141].

Somewhat contradictory studies have reported the effects of nitric oxide on detrusor contractility. Several reports have shown that nitric oxide can act in an inhibitory fashion on bladder smooth muscle (Figure 5), inhibiting contraction and/or inducing relaxation not only in the detrusor but also in the urethra [33, 142, 143]. On the other hand, others have seen that nitric oxide contrarily can increase bladder contractility during electrical field stimulation [144], possibly by aiding intracellular Ca^2+^ release in myocytes (Figure 5) [145, 146]. Some studies performed on other tissues have indicated that nitric oxide can facilitate vesicular release of ACh and ATP [147, 148]. This facilitation of vesicular release of ACh and/or ATP has been suggested to be responsible for the main part of the contractile effect of nitric oxide in the bladder. However, the occurrence of such a mechanism still remains to be fully proven.

One must also point out the existence of different sites of nitric oxide production in the bladder [149]. A recent study by Lee et al. [150] indicates that urothelial nitric oxide formed by iNOS, apart from being the isoform responsible for the inflammatory and apoptotic effects of nitric oxide, might sensitize bladder afferent nerve terminals and thereby increase bladder contractility, while nitric oxide formed via eNOS might have an inhibiting effect on bladder contraction (Figure 5). Since reports have shown both relaxatory and facilitatory effects of nitric oxide, as well as direct effects on the detrusor and modulatory effects via afferent nerves, and that these effects can be altered during disease, the idea of nitric oxide acting in different fashion on bladder contraction due to different conditions seems to be established.

## 6. Conclusion

Evidence about the importance of the signalling molecules in the urinary bladder functioning in health and disease is growing rapidly. Urothelial signalling has a major part in the precisely tuned machinery of the sensory web within urinary bladder wall, which not only enables bladder fullness sensation and proper micturition but also is involved in a wide variety of bladder disorders. These disorders are ranging from asymptomatic, irritative bladder discomfort to life-threatening haemorrhagic cystitis and bladder cancer. Despite many contradictions, as well as unclear and insufficient data, it is crucial to acknowledge the meaning and impact of urothelial signalling molecules on various aspects of the urinary bladder action. This must be taken into account, especially in sight of future research and the development of more effective disease management.

## Figures and Tables

**Table 1:** Signalling molecules and receptor expression in urothelial cells.

Signalling molecule	Receptor	Method of detection
*Α*Ch	Muscarinic (M1–M5)	IHC, IB [48] RT-PCR [151]

ACh	Nicotinic (subunits alpha 3, 5, 7, 9, and 10 and beta 3 and 4)	RT-PCR [37, 41]

Adenosine	Purinergic (P1 or AR-A1, A2a, A2b, and A3)	IB [96, 102]

ATP	Purinergic (P2X_1–7_ and P2Y_1,2,4_)	ICC, IB, and PCR [152] RT-PCR [78]

A/NA	Adrenergic (*α*_1A_, *α*_1D_, and *β*_1–3_)	IB, IHC [153] ISH [154], RT-PCR [130]

VIP/PACAP	PAC1, VPAC1, and VPAC2	ICC [155] RT-PCR [156]

EGF	EGFR	IHC [157]

Substance P	Tachykinin (NK1)	IB, IHC, and RT-PCR [158]

Bradykinin	Bradykinin (B2)	ICC, RT-PCR [159]

Display of a selection of signalling molecules and their corresponding receptors shown to be expressed in the urothelium, as well as the methods used for receptor detection. ACh: acetylcholine; ATP: adenosine 5′-triphosphate; A: adrenaline; NA: noradrenaline; VIP: vasoactive intestinal peptide; PACAP: pituitary adenylate cyclase activating polypeptide; PAC: pituitary adenylate cyclase; VPAC: vasoactive intestinal peptide receptor; EGF: epidermal growth factor; EGFR: epidermal growth factor receptor; RT-PCR: reverse transcriptase polymerase chain reaction; IHC: immunohistochemistry; IB: immunoblotting; ICC: immunocytochemistry; ISH: in situ hybridization.
